# The Irritation of House Dust: DEHP Heightens Inflammatory Response in Allergy Sufferers

**Published:** 2008-11

**Authors:** Carol Potera

Past research has suggested that di(2-ethylhexyl) phthalate (DEHP), a commonly used plasticizer, contributes to asthma symptoms in children [*EHP* 116:98–103 (2008)] and to dermatitis caused by dust mite allergens in mice [*EHP* 114:1266–1268 (2006)]. Both the prevalence of allergic diseases and environmental exposure to phthalates have increased dramatically in the past several decades, but few studies have examined how people’s mucosal airways respond to inhaled DEHP. A new study reveals that exposure to DEHP in house dust altered the response of nasal mucosa in allergic people but not in nonallergic people **[*EHP* 116:1487–1493; Deutschle et al.]**.

DEHP is found in polyvinyl chloride pipes, flooring, food containers, and other household products. Oral intake is the main route of exposure, but inhalation offers an alternative route. DEHP vaporizes from consumer products directly into the home and attaches to inhalable airborne dust particles.

The subjects included 16 controls and 16 people who were allergic to house dust mites. The researchers exposed the subjects to one of two house dust samples—vacuumed samples containing 0.41 mg/g (DEHP_low_) or augmented samples containing 2.09 mg/g (DEHP_high_)—for 3 hours. Nasal fluid was collected after exposure to measure biomarkers of allergic inflammation, including interleukin (IL)-2, -4, -5, -6, and -8, granulocyte colony–stimulating factor (G-CSF), and eosinophilic cationic protein (ECP). The expression of 1,232 genes was analyzed by microarrays in biopsies obtained from one nostril.

Following either DEHP dose, the nonallergic group experienced no changes in nasal mucosa, and biomarker levels did not change significantly. However, DEHP exposure was associated with biomarker changes in the allergic group: half the allergic subjects challenged with DEHP_low_ showed significantly elevated levels of G-CSF, ECP, IL-5, and IL-6, whereas other allergic subjects exposed to DEHP_high_ showed significantly diminished levels of G-CSF and IL-6, suggesting a reduced immune response.

DEHP is a known modulator of gene expression, as illustrated by the current study results. Among healthy subjects, between the two exposure groups, 6 genes were upregulated and 4 were down-regulated. Among allergic subjects, between the two exposure groups, 8 genes were upregulated and 8 were downregulated. One of the genes elevated by DEHP was anti-Müllerian hormone, which is associated with proper gonad development in males. DEHP dampened the expression of lactate dehydrogenase A and fibroblast growth factor 9, regulators of testis formation. Although the gene expression data shed little light on allergic reactions, they support earlier evidence for phthalates’ action as endocrine disruptors that can impair reproductive tract development.

